# Lifestyle patterns, nutritional, and metabolic syndrome determinants in a sample of the older Iranian population

**DOI:** 10.1186/s12877-024-04659-1

**Published:** 2024-01-08

**Authors:** Ali Dehghani Ahmadabad, Leila Jahangiry, Neda Gilani, Mahdieh Abbasalizad Farhangi, Eesa Mohammadi, Koen Ponnet

**Affiliations:** 1 Department of Geriatric Health, Faculty of Health, Tabriz University of Medical Sciences, Tabriz, Iran; 2 Department of Health education and promotion, Faculty of health, Tabriz University of Medical Sciences, Tabriz, Iran; 3 Department of Statistics and Epidemiology, Faculty of Health, Tabriz University of Medical Sciences, Tabriz, Iran; 4 Drug Applied Research Center, Tabriz University of Medical Sciences, Tabriz, Iran; 5 Nursing Department, Faculty of Medical Sciences, Tarbiat Modares University, Tehran, Iran; 6 Faculty of Social Sciences, Imec-Mict-Ghent University, Ghent, Belgium

**Keywords:** Metabolic syndrome, Lifestyle, Older adults, Factor analysis

## Abstract

**Background:**

Chronic diseases and metabolic disorders are prevalent health concerns that often escalate with increasing age and thus affect older individuals. The proportion of the elderly population in Iran increased from 7.22% in 2006 to 12.0% in 2023. The current study aimed to evaluate lifestyle patterns and lifestyle risk factors among patients with metabolic syndrome (MetS) based on dietary, physical activity, and smoking, as well as MetS components.

**Methods:**

This cross-sectional study included 582 older people with MetS living in Yazd, Iran. Latent class analysis (LCA) was used to determine the lifestyle behaviors of diet patterns, smoking, and physical activity. Dietary intake was measured using a validated food frequency questionnaire, and dietary patterns were identified using principal component analysis (PCA). Clinical measurements of MetS components were examined using relevant guidelines.

**Results:**

The mean age of the participants was 72.71 years (SD = 5.57). Using PCA, two dietary patterns were identified: traditional patterns (e.g., fruits, fish, poultry, vegetables, meats, salt, and sugar sweetened beverages) and high-fat patterns (e.g., high-fat dairy). Applying LCA identified two classes of lifestyle patterns. About 35% (*n* = 204) of the participants were categorized in a low-risk class (I) and characterized by physical activity (0.93%, *n* = 190), a traditional pattern for diet (61%, *n* = 122), and zero probability of smoking. About 65% (*n* = 378) of the patients were categorized in high-risk class (II) and characterized by low physical activity levels (69%, *n* = 261), cigarette smoking (71.6%, *n* = 271), and a high-fat dietary pattern (56.9%, *n* = 215).

**Conclusion:**

The results of our study indicated two distinct classes within the patients. In class I, aging patients with MetS exhibited characteristics such as engagement in physical activity and having a traditional pattern for diet. Class II, with a higher prevalence of lifestyle risk factors, included individuals who engaged in cigarette smoking, displayed low physical activity (69%), and having a high-fat diet. The combination of these lifestyle factors exposed them to a heightened risk of developing MetS. The findings could guide healthcare professionals to be aware of the associations between different lifestyle risk factors and to focus on multiple behaviors at the same time.

## Background

The World Health Organization estimates that the number of people older than 60 years in the world will increase from 1.2 billion in 2025 to more than 2 billion by 2050 [1]. Iran is rapidly becoming an aging society and has one of the fastest growth rates of the older population in the world [2]. The proportion of the elderly population in Iran increased from 7.22% in 2006 to 12.0% in 2023. It is expected that the elderly population will increase to 30.4% by 2050 [3]. Chronic diseases and metabolic disorders are prevalent health concerns that often escalate with advancing age, disproportionately affecting older individuals [4]. These conditions, such as heart disease, diabetes, and hypertension, are characterized by long-lasting and recurring health issues that require ongoing management. As individuals age, their bodies become more vulnerable to these ailments due to a combination of factors, including physiological changes, lifestyle habits, and genetic predispositions [5]. Risk factors, such as sedentary lifestyles, poor dietary choices, obesity, smoking, and a decline in physical activity, further contribute to the development and progression of these conditions in older adults [6]. Consequently, older people bear a heavier burden of chronic diseases and metabolic disorders, necessitating comprehensive healthcare strategies and support systems tailored to them [7]. It has been recognized that metabolic syndrome (MetS) increases the occurrence of cardiovascular disease (CVD) and doubles the risk of developing cardiovascular disorders, increases the risk of developing diabetes by five times, and increases the odds of death by 20–80% [8, 9].

Several definitions have been presented for the diagnosis of MetS [10]. The criterion provided by the adult treatment panel (ATP III) is commonly used in clinical assessments. According to the ATP III definition, the presence of at least three of the five components of hypertension, central obesity, impaired fasting blood sugar, high triglycerides, and a reduction in high-density lipoprotein (HDL) indicate MetS [11, 12].

Epidemiological studies have demonstrated that the prevalence of MetS has increased worldwide and varies from 12 to 37% among the Asian population and from 12 to 26% in developed countries [13–15].

Iran has one of the highest rates of MetS prevalence. A systematic review and meta-analysis revealed that the pooled prevalence of MetS among Iranian adults is alarmingly high (43%) [16]. Another study reported that the overall prevalence of MetS was 37.05%, with a higher prevalence in women (49.8%) than in men (24.3%) [4].

Lifestyle factors play a significant role in the development and management of MetS, highlighting the importance of healthy habits, such as regular physical activity, a balanced diet, and weight control [17]. Several studies have demonstrated that engaging in moderate physical activity, such as regular walking, can significantly reduce the risk of MetS and help prevent its onset. Physical activity plays a crucial role in maintaining overall health and well-being, particularly in relation to cardiovascular health and metabolic function [18]. Regular walking, which is an accessible and low-impact form of exercise, has been associated with numerous benefits for cardiovascular health [19]. It can help lower blood pressure and improve lipid profiles by increasing HDL, reducing triglyceride levels, and enhancing insulin sensitivity [20]. These positive effects on cardiovascular health contribute to a decreased risk of developing metabolic syndrome, a cluster of conditions that includes abdominal obesity, high blood pressure, elevated blood sugar levels, and abnormal lipid levels. By incorporating moderate physical activity, such as regular walking, into daily routines, individuals can improve their cardiovascular health, reduce the risk of cardiovascular diseases, and mitigate the development of metabolic syndrome [21, 22].

Various social and demographic factors, such as education level, socioeconomic status, marital status, and cultural background, can significantly influence the choices and behaviors of older individuals [23]. These factors shape the social and environmental contexts in which they live and interact, consequently impacting their lifestyle choices. For instance, older adults with higher education levels and better socioeconomic status may have access to resources, knowledge, and opportunities that enable them to adopt healthier lifestyles [24, 25]. They may engage in regular physical activity, follow balanced diets, and have better overall health outcomes. On the other hand, elderly individuals with lower education levels or disadvantaged socioeconomic backgrounds may face barriers to adopting healthy lifestyles, such as limited access to nutritious food options or opportunities for physical activity [26]. Moreover, cultural and ethnic backgrounds can also influence lifestyle patterns among the elderly, as different groups of communities have different lifestyle patterns [27].

One approach to evaluating lifestyle patterns is latent class analysis (LCA) [28]. LCA is a person-centered analytic approach to categorize individuals according to the maximum probability of being in a class [29]. LCA categorizes patients based on their observed characteristics or indicators, aiming to identify unobserved latent classes that explain the patterns of responses or behaviors observed in the data. It provides a data-driven approach to understanding heterogeneity within a population and helps identify meaningful subgroups with similar characteristics. LCA allows us to identify and account for heterogeneity by grouping individuals into latent classes based on shared characteristics. By categorizing older patients into low- and high-risk classes, LCA helps in risk stratification, which is essential for effective healthcare management. It allows healthcare providers to identify patients who are at higher risk of adverse outcomes or complications and allocate appropriate resources and interventions accordingly. It helps in understanding the factors that contribute to the classification and can assist in predicting future outcomes or assessing the effectiveness of different interventions for each risk class.

The outcomes of LCA include the number of latent classes and the probability of each indicator in each class. Membership in each class is based on similarities in responses to factors related to a set of observed behaviors [29].

Applying an LCA approach to the study of lifestyle risk factors in the elderly population with MetS can provide valuable insights into the diverse range of lifestyles and behaviors that contribute to the development and progression of MetS. Researchers can identify subgroups characterized by specific combinations of lifestyle factors, such as physical activity levels, dietary habits, and smoking status [23]. This information can provide a better understanding of how lifestyle choices interact with and influence metabolic health outcomes [30, 31]. One of the advantages of the LCA method is that multiple features that categorize individuals into subgroups can be examined simultaneously instead of examining each of these features separately [32]. Furthermore, by recognizing latent classes, researchers can design targeted interventions and personalized strategies tailored to the unique needs of each subgroup, optimizing the effectiveness of preventive and therapeutic interventions.

The current study aims to evaluate lifestyle patterns and latent classes of lifestyle risk factors among patients with MetS based on diet, physical activity, smoking, and MetS components. This study adds to the literature due to its use of LCA to investigate lifestyle patterns and their association with MetS determinants in the older Iranian population. While the factors’ associations with MetS are well known, this study adds a new perspective by identifying distinct lifestyle patterns within the aging population and examining how these patterns relate to the determinants of MetS. This approach offers a more nuanced understanding of the interplay between lifestyle, nutrition, and MetS in older Iranians, potentially uncovering insights that previous research may not have captured.

## Methods

### Study design, participants, and sampling

**Fig. 1:**
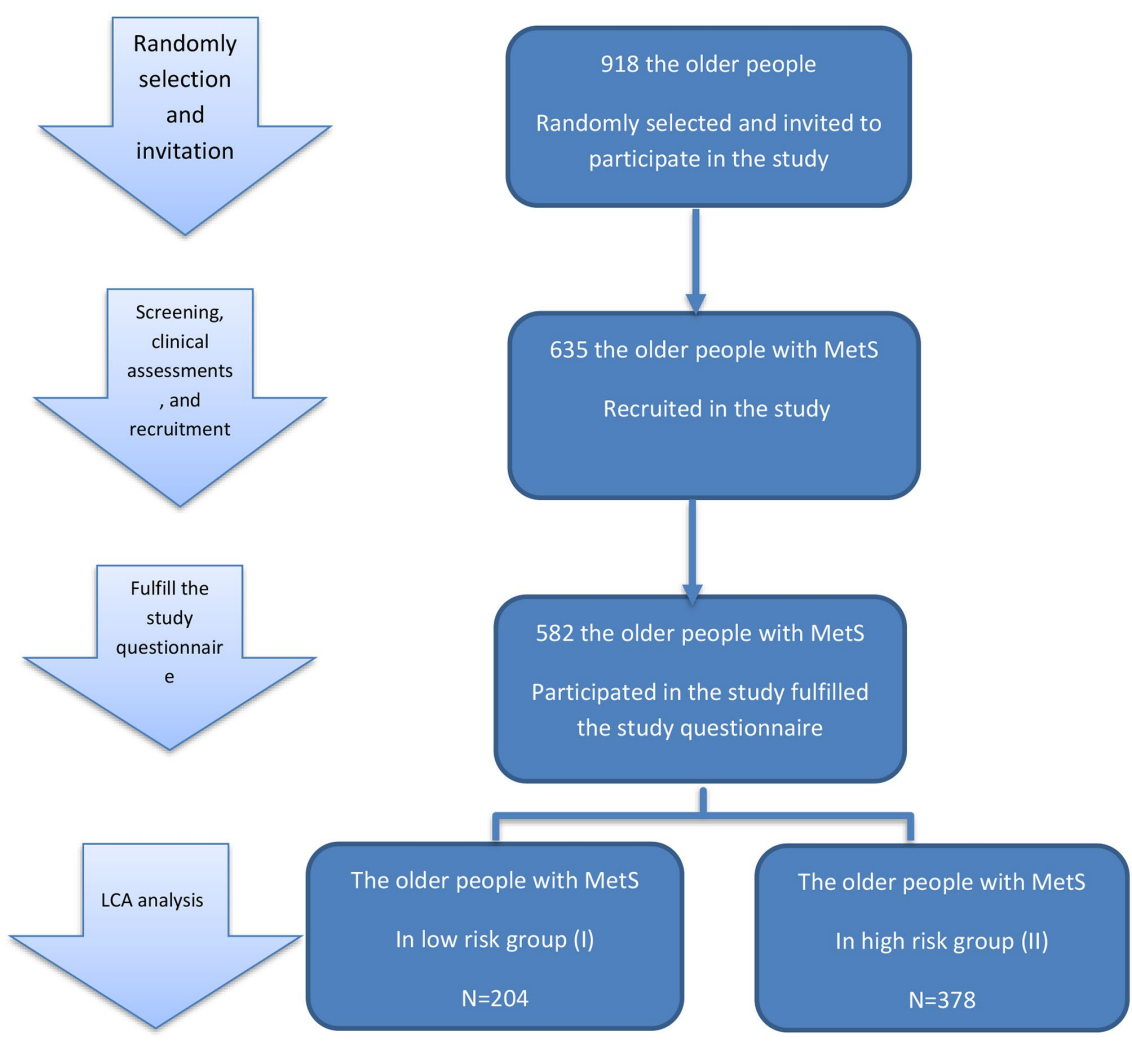
Study selection process

This cross-sectional study was conducted in Yazd, Iran, a city with an estimated urban population of 563,076 people and an aging urban population (60 years and over) of 77,625 people [33]. Information about this population is accessible through an integrated electronic health system (SIB) in Iran. This system contained comprehensive health records for individuals in health centers. Therefore, 10 health centers were randomly selected from a total of 28 total health centers in Yazd. This random selection helps ensure a representative sample from the overall population. The main researcher (AD) utilized the SIB system to extract a list of individuals aged 60 years and over from each of the selected health centers. A total of 918 eligible people from Yazd urban primary health care centers were randomly selected using online software (www.random.org) and invited to participate in the study. During the phone invitation, a trained health researcher contacted the eligible and interested individuals. They explained the study, its purpose, and invited them to health care centers for clinical assessments and screening for MetS. This step was essential to ensure that participants understand the study requirements and procedures. Individuals who expressed interest in participating were asked to visit health care centers for clinical assessments and screening for MetS. From the initially invited 918 individuals, a total of 635 patients diagnosed with MetS completed the clinical assessments and screening. The enrolled participants were then provided with a study questionnaire and 582 of them completed it (See the recruitment procedure in Fig. 1). The study was conducted in the first semester of 2022.

The inclusion criteria were (a) having an age of 60 years and over, (b) living in Yazd city, and (c) having an interest in participating in the study. The exclusion criteria were (a) having been diagnosed with cognitive disorders, (b) following a special dietary lifestyle, and (c) having movement restriction. Data were collected using interviews, physical examinations, and laboratory measurements. Before participating in the study, informed written consent was obtained from all the participants. All of the methods of the current study were performed in accordance with the relevant guidelines and regulations.

### Sample size

LCA can be considered a “large sample” method; with sample sizes greater than 500, models and fit statistics have been shown to be highly accurate [34]. With smaller sample sizes, particularly when *N* < 300, the results are less reliable [35].

### Demographic and anthropometric variables

Data were collected using three questionnaires. A short self-developed questionnaire collected demographic data related to age, gender, marital status (single, married, divorced, or widowed), highest educational qualification (illiterate, primary or secondary education, or university degree), income level, and religion (Muslim or Zoroastrian). MetS was defined based on the national cholesterol education program (NCEP) adult treatment panel (ATP III) as the presence of three or more criteria of five metabolic syndrome criteria except for waist circumference, which was determined as ≥ 90 cm for both genders for the aging Iranian population [36]: systolic or diastolic blood pressure 130/85 mmHg or higher, fasting blood glucose level 100 mg/dl or higher, triglycerides level over 150 mg/ dl, HDL level < 40 mg/dl in men and < 50 mg/dl in women. All measures related to anthropometric indicators were performed in one day for each participant by an expert researcher. Blood pressure was measured using a digital arm sphygmomanometer (made by Omron, model 7 M from Vietnam) after at least 10 min of rest, sitting and measuring it from the dominant hand of the participant in two stages with an interval of 5 min, and recording their mean. Blood sampling was done by 3 ml of brachial vein blood per person after recommended fasting for 10–12 h. Body weight was measured with minimum clothes and without shoes, with an accuracy of 0.1 kg, using a digital scale made in Germany (DLT-411 model). Height was measured with a constant tension tape with an accuracy of 0.5 cm in a standing position without shoes. Body mass index (BMI) was calculated by dividing the person’s weight in kilograms by the square of height in meters [37]. Waist circumference was measured at the midpoint between the iliac blade and the lowest expiratory rib using an inflexible tape meter [37].

### Dietary intake assessment

Data on dietary intake were measured using a validated semi-quantitative 147-item food frequency questionnaire (FFQ) completed by an expert nutritionist and originally developed for the Tehran Lipid and Glucose Study (Research Institute for Endocrine Sciences in Iran) [38]. This questionnaire has been adapted to the Iranian context. Each participant was asked to calculate how many frequencies of each food they used during the last year on a monthly, weekly, annual, or daily basis. The portion sizes were converted to grams using a household scale.

### Physical activity (PA) assessment

The International Physical Activity Questionnaire (IPAQ), a well-validated questionnaire, was employed in this study to classify individuals into categories of low or high physical activity [39]. Based on the information provided by the respondents, the total physical activity score was calculated by walking and moderate- and vigorous-intensity activities performed each week, as well as by the time spent on them [40]. The classification was based on a cutoff score of 600 metabolic equivalent of task (MET)-minutes per week. Specifically, participants scoring below 600 MET-minutes per week were categorized as having low physical activity levels, while those scoring 600 MET-minutes per week or above were classified as having high physical activity levels. This cutoff score of 600 MET-minutes per week is commonly used to distinguish between different levels of physical activity and was utilized as a benchmark in this study to classify participants into low or high physical activity groups [41, 42].

### Smoking and opium addiction

A question about smoking (yes/no) and opium use (yes/no) was included in the sociodemographic characteristics’ section, and a binary variable was created for each question.

### Statistical analysis

Baseline characteristics of participants were expressed as means (SD) for continuous variables and frequencies and percentages for categorical variables. Principal component analysis (PCA) was used based on 17 (derived from FFQ [38]) food groups to extract major dietary patterns. PCA is a method performed to decrease a set of features [43]. Factor analysis with varimax rotation was used to develop an interpretable solution. Varimax rotation was used to minimize the number of components. Eigenvalues greater than 1 were considered to determine the number of factors and factor interpretability [44]. Two dietary factors were derived by considering data reduction in the factor analysis. By interpreting the data and considering the previous literature [23, 45], the derived dietary patterns were labeled. The factor score for each pattern was calculated by summing the consumption of all food groups weighted by their factor loadings, and for each dietary pattern, the participants received a factor score.

LCA [29] was used to identify clusters of behaviors among the participants (based on binary indicators of lifestyle risk factors [cigarette smoking, low physical activity, and a non-healthy diet]). An LCA model was performed using three indicator variables: physical activity (two response categories), dietary patterns, and smoking. Latent class regression was applied using age, gender, marital status, metabolic syndrome components, education, and income. The poLCA package for R (version 3.4.2) was used to fit the latent class models to estimate the model [46]. To select the number of clusters that best fit the data, we first fit a two-class model and increased the number of classes one by one up to a six-class model. Models were compared using the Akaike information criterion (AIC), Bayesian information criterion (BIC), likelihood ratio/deviance statistic (GSq), and Pearson chi-square goodness of fit statistic (Chisq). The best model was selected based on the fit statistics (with lower values for AIC and BIC, upper values for Gsq, and Chisq values indicating better fit). Adjusted odds ratios (ORs) of anthropometric, biochemical, and demographic predictors in Class II versus Class I were used to investigate the factors related to being in classes of patients with MetS.

## Results

### Sample characteristics

The participants’ descriptive characteristics are presented in Table 1. This cross-sectional study included 582 elderly patients with MetS. The mean age of the participants was 72.71 years (SD = 5.57), and BMI was 28.13 (4.37) kg/m^2^. The majority of participants were women (55%), married (72.8%), and Muslim (91.7%) and had a primary education (46.3%).

**Table 1:** Baseline characteristics of the study participants with metabolic syndrome based on ATP III definition

	Total (*n* = 582); Mean (SD)
**Age**	72.71 (5.57)
**Gender** N (%)	
Female	320 (55)
Male	262 (45)
**Marital status** N (%)	
Married	424 (72.8)
Widowed/divorced/single	158 (27.1)
**Education** N (%)	
Illiterate	193 (33.1)
Primary education	270 (46.3)
Secondary education	85 (14.6)
University and higher	34 (6)
**Income Rial**	
< 50,000,000	259 (44.4)
50,000,000 − 10,000,000	271 (46.5)
> 10,000,000	52 (9.1)
**Religious** N (%)	
Muslim	534 (91.7)
Zoroastrian	48 (8.3)
**Smoking (yes)**	114 (19.5)
**Opium addiction (yes)**	25 (4.2)
BMI (kg/m^2^)	28.13 (4.37)
WC (cm)	99.86 (10.66)
SBP (mmHg)	148.59 (15.14)
DBP (mmHg)	85.15 (6.12)
CHO(mg/dl)	203.69 (41.94)
HDL-C (mg/dl)	37.75 (10.25)
TGs (mg/dl)	210.29 (58.28)
FBS (mg/dl)	151.65 (44.28)

Note: BMI: Body mass index; WC: waist circumference; SBP: systolic blood pressure; DBP: diastolic blood pressure; CHO: cholesterol; HDL-C: high-density lipoprotein; TGs: triglycerides; FBS: fast blood sugar

### Findings of the factor analysis

Two dietary patterns were extracted using a factor analysis method, and the factors were labeled according to the food groups with high-loading traditional patterns for diet and high-fat patterns, which explained 10.61% of the variance. The traditional pattern was highly correlated with fruit, fish, poultry, vegetables, meats, whole grain, salt, tea, coffee, sweetened beverages, sauces, nuts, seeds, liquid oil, refined grains, eggs, and discretionary snack food. High-fat patterns demonstrated high factor loadings for high-fat dairy products and solid fats (Table 2).

**Table 2:** Factor loading matrix for extracted major dietary patterns

Food groups	Traditional pattern for diet	High fat pattern
Fruit	0.700	− 0.133
Fish and poultry	0.631	
Vegetables	0.624	
Meats	0.583	0.167
Whole grain	0.567	− 0.128
Salt	0.491	
Tea and coffee	0.473	
Sugar sweetened beverage	0.468	
Souce	0.464	− 0.174
Nuts and seeds	0.402	− 0.176
Liquid oil	0.370	-
Refined grains	0.353	0.168
Egg	0.295	-
Discretionary snack food	0.255	− 0.119
High fat dairy products	-	0.889
Solid fat	− 0.364	0.280
Low fat dairy products	-	− 0.864

### Results of the latent class analysis

Table 3 shows the information criteria for a two-class model of LCA by performing the iterative process. The table includes the number of latent classes, predicted class memberships (by modal posterior prob), and the conditional probabilities of having risk factors for each class. The latent class prevalence and the probability of item response for each indicator are presented in Table 4. According to our findings, about 35.1% (*n* = 204) of the participants were categorized in a low-risk class (I) and characterized by physical activity (0.93%, *n* = 190), a traditional pattern for diet (61%, *n* = 122), and zero probability of smoking. About 65% (*n* = 378) of the patients were categorized in high-risk class (II) and characterized by low physical activity levels (69%, *n* = 261), cigarette smoking (71.6%, *n* = 271), and high-fat dietary patterns (56.9%, *n* = 215).

**Table 3:** Information criteria of models with different numbers of classes

	Number of Classes				
Information Criterion	2	3	4	5	6
AIC	2323.446	2329.524	2334.788	2343.318	2352.950
BIC	2362.744	2390.655	2417.751	2448.113	2479.578
Gsq	10.93185	7.010328	0.8039911	0.8039911	0.4363142
Chisq	8.800808	5.956545	0.7294985	0.7294985	0.3755706

AIC: Akaike Information Criterion; BIC: Bayesian Information Criterion; GSq: Likelihood ratio/deviance statistic; Chisq: Pearson Chi-square goodness of fit statistic

**Table 4:** The two latent classes models of lifestyle

	Class I (Low risk) (*n* = 204)	Class II (high risk) (*n* = 378)
Latent class prevalence ^a^	0.3505	0.6495
**Variables**		
Cigarette smoking		
Yes	0.0000	**0.7167**
No	1.0000	0.2833
Low physical activity (< 600 MET-min/week)		
Yes	0.0624	**0.6923**
No	0.9376	0.3077
Diet		
Traditional pattern	0.6163	0.4307
High fat pattern	0.3837	0.5693

Note. The values show the conditional probabilities of having risk factor for each group. a: Predicted class memberships (by modal posterior prob.)

The conditional probabilities of a “Yes” response to each risk behavior are listed in Table 4. The possibility of a “No” response was calculated by subtracting the item response probabilities from 1. Class (I) includes 204 individuals with a mean age of 72.20 ± 5.50 years and is characterized by a high level of physical activity and a traditional diet. In contrast, class (II) includes 378 patients with a mean age (72.98 ± 5.59) and is characterized by a high-fat dietary pattern, low level of physical activity, and high cigarette smoking level (Fig. 1).

The adjusted ORs of anthropometric, biochemical, and demographic predictors in Class II versus Class I are reported in Table 5. Significant determinants for the unhealthy lifestyle of aging patients with MetS are SBP (mmHg), DBP (mmHg), TG (mg/dl), gender (man), status as widow/divorced/single, cigarettes smoking, and opium addiction. The results indicate that the OR (standard error) of membership in class II as an unhealthy lifestyle in comparison with class I increased with SBP (OR 1.13, SE = 0.78; *p* = 0.019), DBP (OR 1.10, SE = 0.95; *p* = 0.044), and TG (OR 1.35, SE = 1.00; *p* < 0.001). Compared to women, men are 3.94 times more likely to engage in an unhealthy lifestyle (class II) than women (OR 3.94, SE = 1.20; *p* < 0.001). Widowed/divorced or single participants are 1.18 times (OR 1.18, SE = 0.98; *p* ≤ 0.001), participants with smoking are 2.34 times (OR 2.34, SE = 1.06; *p* = 0.027), and opium addicted participants are 3.26 times (OR 3.26, SE = 1.11; *p* = 0.003) in higher risk to categorize in unhealthy lifestyle. Additionally, higher education level has an inverse association with engaging in an unhealthy lifestyle (≤ high school, OR 0.75, SE = 0.002; *p* < 0.001, high school degree, OR 0.83, SE = 0.003; *p* < 0.001, and college degree OR 0.96, SE = 0.002; *p* < 0.001).

**Table 5:** Adjusted Odds Ratio of Anthropometric, biochemical and demographic predictors in Class II versus Class I

Predictors	Class II versus Class I	
	Adjusted Odds Ratio (Standard Error)	*P*-value
Age (year)	1.79 (0.77)	**0.068**
BMI (kg/m^2^)	4.75 (4.04)	0.316
WC (cm)	3.38 (3.19)	0.292
SBP (mmHg)	1.13 (0.78)	**0.019**
DBP (mmHg)	1.10 (0.95)	**0.044**
TG (mg/dl)	1.35 (1.00)	**< 0.001**
TC (mg/dl)	0.88 (1.14)	0.377
LDL-C (mg/dl)	1.04 (1.02)	0.089
HDL-C (mg/dl)	0.98 (1.02)	0.588
FBS (mg/dl)	1.00 (1.01)	0.190
**Gender**		
Female	Ref	
Male	3.94 (1.20)	< 0.001
**Education**		
Illiterate	Ref	
<=High School	0.75 (0.002)	< 0.001
Diploma	0.83 (0.003)	< 0.001
College degree	0.96 (0.002)	< 0.001
**Marital Status**		
Married	Ref	
Widow	1.18 (0.98)	< 0.001
Divorced/not married	1.02 (0.99)	0.303
Income		
1	Ref	
2	0.74 (0.66)	0.263
3	0.87 (0.69)	0.208
4	1.02 (0.98)	0.298
Smoking		
Yes	2.34 (1.06)	**0.027**
No	Ref	
Opium addiction		
Yes	3.26 (1.11)	**0.003**
No	Ref	

Note: BMI: Body mass index; WC: waist circumference; SBP: systolic blood pressure; DBP: diastolic blood pressure; CHO: cholesterol; HDL-C: high-density lipoprotein; TGs: triglycerides; FBS: fast blood sugar

## Discussion

In this study, we examined the lifestyle patterns of aging people with MetS. Our study revealed that the participants could be classified into two subclasses according to their nutritional patterns, smoking, and physical activity. In addition, two dietary patterns (traditional pattern and high-fat pattern) were identified using factor analysis. Aging people with MetS in class I (low risk) had the following characteristics: physical activity (0.93%) and a traditional pattern for diet (61%). Class II (high risk), with a higher prevalence of lifestyle risk factors, reflects a high prevalence of cigarette smoking (71%), low physical activity (69%), and a high-fat diet (56%) as the dominant dietary pattern.

Previous research has consistently demonstrated the benefits of considering the co-occurrence of unhealthy lifestyle behaviors as an effective approach for disease prevention. Specifically, studies have focused on examining combinations such as smoking and low physical activity [47] as well as smoking and low consumption of vegetables and fruits [23, 48]. By examining the cluster of behaviors together, researchers can identify a synergistic effect in which the negative impact on health is amplified when these behaviors coexist [49, 50]. A Chinese study by Zhang et al. examined a sample of individuals aged 65–105 from the 2011–2012 Chinese Longitudinal Healthy Longevity Survey dataset and identified four distinct health lifestyle patterns among older adults. These four categories included one characterized by consistent engagement in healthy behavior (22.4% of the sample), another marked by smoking and drinking issues (22.1%), a third with sleep problems (20.2%), and a fourth classified as the sedentary group (34.6%). Notably, the largest group was the sedentary category, representing over one-third of the studied population, while only one-fifth of the participants exhibited healthy lifestyles. The majority of respondents had challenges related to sleep, alcohol, or smoking. In general, the health lifestyle profile of older Chinese adults appeared less promising [51]. Another study in Spain used LCA and identified three distinct multimorbidity patterns among Spanish adults aged 50 and older based on the presence or absence of 11 chronic conditions. These patterns were associated with various health outcomes at baseline and remained significant predictors of these outcomes over a three-year follow-up period, contributing valuable insights into the understanding of multimorbidity in this population, in contrast to previous studies with varying results regarding the number of identified clusters [52].

In our study, we examined aging patients with MetS and found a significant association between their dietary patterns, physical activity levels, and risk of MetS. Specifically, individuals in class II displayed a high-fat dietary pattern combined with low physical activity and a high level of smoking. This combination of factors exposed them to a higher risk of developing MetS. A high-fat diet, characterized by an excessive intake of unhealthy fats, can contribute to weight gain, dyslipidemia, and insulin resistance, all of which are key components of metabolic syndrome [53, 54]. Additionally, a low level of physical activity further exacerbates these risks by impairing metabolic function, reducing energy expenditure, and promoting sedentary behaviors [45, 55]. Our findings highlight the importance of addressing both dietary habits and physical activity levels in the management and prevention of MetS among aging patients, emphasizing the need for comprehensive lifestyle interventions to mitigate the associated risks. By addressing multiple unhealthy behaviors simultaneously, healthcare professionals and policymakers can develop more comprehensive and impactful initiatives to promote healthier lifestyles and improve public health outcomes [49, 50].

The results of our study revealed two distinct dietary pattern included the traditional dietary pattern and high fat pattern. Patients in the lower risk group (class I) exhibited a traditional dietary pattern characterized by the range of food groups encompasses fruits, fish, poultry, vegetables, meats, whole grain, salt, tea, coffee, sweetened beverages, sauces, nuts, seeds, liquid oil, refined grains, eggs, and discretionary snack food. In contrast, patients in the higher risk group (class II) were characterized by a high-fat dietary pattern. This pattern was marked by a correlation with high-fat dairy products and solid fats. Our findings align with a study by Stefler that identified a traditional dietary pattern was associated with all-cause and cause-specific mortality outcomes in Eastern European populations [56]. A longitudinal cohort study that confirmed the positive association between a traditional Mediterranean dietary pattern (including fruit, vegetables, and fish) and individuals mortality within the study population [57]. Additionally, another study highlighted that a traditional pattern characterized by greater intakes of red meat and potatoes and lesser intakes of low-fat dairy and fruit was associated with higher blood pressure, higher concentrations of HDL cholesterol, total cholesterol, and glucose [58]. This suggests that while certain components of the traditional dietary pattern, notably fruits and vegetables, are recognized for their health benefits, the impact of other elements on MetS in the aging population is nuanced. The consumption of meats, particularly red and processed meats, has been associated with an increased risk of MetS and diabetes due to the presence of saturated fats that may contribute to insulin resistance [59]. It is likely that certain aspects of traditional diets, such as high salt and sugar intake, may contribute to an elevated risk of MetS, especially in the aging population. The incorporation of salt and sweets can lead to various health issues, including hypertension, insulin resistance, and obesity, which are key components of MetS [58, 59].

Patients in the higher-risk group (class II) exhibited a distinctive dietary pattern characterized by a higher fat intake. This dietary profile was notable for its correlation with the consumption of high-fat dairy products and solid fats. These dietary choices are significant indicators of a pattern associated with elevated fat content, which, in turn, has implications for metabolic health. The heightened presence of high-fat dairy and solid fats in the diet of these patients suggests an association with increased components of metabolic syndrome. The observed correlation suggests that the dietary habits within this higher-risk group may play a contributory role in the pathogenesis of MetS [60].

Furthermore, the findings of the regression analysis showed that aging patients with MetS in class II were significantly at higher risk by increasing systolic blood pressure (1.13 times), diastolic blood pressure (1.10 times), and TG (1.35 times) than patients in class I. Aging men with MetS were significantly 3.94 times higher risk than women to be clustered in the high risk groups, and patients with high-level education were less likely to be clustered in class II.

### Limitations and strengths

The study has both limitations and strengths. One limitation is the cross-sectional design, which hinders the ability to establish causality and determine temporal relationships between variables. Additionally, reliance on self-reported data for dietary patterns and physical activity levels may introduce recall bias and potential inaccuracies. Another limitation is the focus on aging patients with MetS, which may limit the generalizability of the findings to other populations. Although we used random selection as a fundamental principle of sampling, it does not guarantee that the sample accurately reflects the diverse characteristics of the entire aging population. However, the study’s strengths include a well-defined sample of aging patients with MetS, which enhances the internal validity of the findings within this specific population. The inclusion of dietary patterns and physical activity levels provides a comprehensive assessment of lifestyle factors that contribute to MetS. Furthermore, the study’s findings contribute to the existing body of knowledge on the association between high-fat dietary patterns, low physical activity, and the risk of MetS in aging patients, highlighting the importance of targeted interventions in this vulnerable population.

## Conclusion

The results of our study indicated two distinct classes within the patients. In class I, aging patients with MetS exhibited characteristics such as engagement in physical activity and having a traditional pattern for diet. Class II, with a higher prevalence of lifestyle risk factors, included individuals who engaged in cigarette smoking, displayed low physical activity (69%), and having a high-fat diet. The combination of these lifestyle factors exposed them to a heightened risk of developing MetS. The findings could guide healthcare professionals to be aware of the associations between different lifestyle risk factors and to focus on multiple behaviors at the same time. These analyses provide important insights into how we might target lifestyle-promoting strategies among older populations with MeTS. Further research can now focus on exploring the specific characteristics and risk factors associated with each class, which may ultimately lead to more targeted and effective strategies for the prevention and management of MetS.

## Data Availability

The data collection tools and datasets generated and/or analyzed during the current study are available from the corresponding author upon reasonable request.

## Abbreviations

MetS: metabolic syndrome
IDF: International Diabetes Federation
AHA: American Heart Association
NHLBI: Heart,Lung,and Blood Institute
CVDs: cardiovascular diseases
HDL-C: high-density lipoprotein cholesterol
NCEP: cholesterol education program
ATP III: adult treatment panel
SPSS: Statistical Package for Social Science
BMI: Body mass index
PA: Physical activity
PCA: Principal Components Analysis
IPAQ: International physical activity questionnaire
FFQ: Food frequency questionnaire
MET: Metabolic equivalent
BIC: Bayesian information criterion
AIC: Akaike information criterion
SD: Standard Deviations
Wc: waist circumference,WHO: World Health Organization
